# Pulmonary fibrosis through the prism of NLRP3 inflammasome: mechanistic pathways and prospective therapeutic innovations

**DOI:** 10.3389/fimmu.2025.1593729

**Published:** 2025-05-05

**Authors:** Mengxue Wang, Yuanyuan Xie, Yuqing Cao, Bing Yu, Qingqing Dai

**Affiliations:** ^1^ Department of Critical Care Medicine, The Obstetrics & Gynecology Hospital of Fudan University, Shanghai, China; ^2^ Department of Critical Care Medicine, The Obstetrics & Gynecology Hospital of Fudan University, Yangtze River Delta Integration Demonstration Zone (QingPu), Shanghai, China; ^3^ Department of Cell Biology, Navy Medical University, Shanghai, China

**Keywords:** NLRP3 inflammasome, pulmonary fibrosis, cellular signaling, inhibitors of NLRP3 inflammasome, therapeutic strategies

## Abstract

Pulmonary fibrosis is a disease that severely affects the patients’ life quality, characterized by lung tissue remodeling and functional impairment. Recent research has found that the NLRP3 inflammasome plays an important role in the pathogenesis of pulmonary fibrosis. Although existing researches have revealed the potential role of NLRP3 in pulmonary fibrosis, many mysteries still remain regarding its specific mechanisms and clinical applications. This article aims to review the mechanisms of action of NLRP3 in pulmonary fibrosis, related signaling pathways, and the latest research progress on its potential as a therapeutic target, in hopes of providing new ideas and directions for future clinical treatment.

## 1 Introduction

Pulmonary fibrosis (PF) is an interstitial lung disease characterized by progressive fibrosis and structural remodeling. Pulmonary fibrosis is categorized into idiopathic and secondary types, both of which are associated with triggers such as environmental exposures, infections, medications, and genetic predisposition. The distinction lies in that the etiology of secondary pulmonary fibrosis is well-established, whereas the underlying causes of idiopathic pulmonary fibrosis (IPF) remain elusive (1–4). As fibrosis progresses, it not only severely affects patients’ quality of life but also increases the risk of early death (5). So far, therapeutic options for pulmonary fibrosis remain limited, as conventional anti-inflammatory agents and immunosuppressants have demonstrated little to no significant efficacy in its treatment (6, 7). Currently, two advanced therapeutic drugs nintedanib and pirfenidone, have been approved for the management of pulmonary fibrosis; Nintedanib is a tyrosine kinase inhibitor that targets vascular endothelial growth factor, fibroblast growth factor, and platelet-derived growth factor signaling pathways. Pirfenidone has antifibrotic, anti-inflammatory and antioxidant effects, although its exact mechanisms are not fully elucidated. However, their efficacy is limited to decelerating the progression of fibrosis, with little potential to achieve a definitive cure (8, 9). In contrast, therapeutics targeting the NLRP3 inflammasome represent a novel approach that directly addresses the inflammatory cascade at its source, potentially offering more comprehensive control of both the inflammatory and fibrotic processes in PF. This innovative mechanism differs fundamentally from current approved therapies by intervening at the level of inflammatory initiation rather than downstream pathway inhibition. Lung transplantation also remains the ultimate treatment method to improve prognosis (8).

The mechanism of pulmonary fibrosis remains complex and mainly involves inflammatory responses, the activation and transformation of fibroblasts, cytokine signaling pathways, oxidative stress, epithelial-mesenchymal transition (EMT), and so on (7). Damage factors cause alveolar epithelial cells to undergo EMT and release pro-inflammatory and pro-fibrotic mediators, which further stimulate the proliferation and differentiation of fibroblasts and the activation of myofibroblasts, ultimately leading to excessive deposition of extracellular matrix (ECM) proteins (10, 11). The inflammatory cascade is central to pulmonary fibrosis pathogenesis, with various cytokines orchestrating this process. In the bleomycin (BLM)-induced pulmonary fibrosis model, alveolar damage leads to the recruitment of inflammatory cells, including macrophages, neutrophils, and lymphocytes. These cells produce key pro-inflammatory cytokines such as IL-1β, IL-6, and TNF-α, which further amplify the inflammatory response. (12). Subsequently, overabundant inflammatory responses release a large number of pro-inflammatory cytokines and growth factors, particularly IL-1β, not only perpetuate inflammation but also directly promote fibroblast activation (10). Furthermore, researches indicate that macrophages play a central role in the development of IPF (13). M1 macrophages induce inflammation by secreting pro-inflammatory cytokines (such as TNF-α, IL-6, and IL-1β), while M2 macrophages promote fibroblast proliferation and differentiation by secreting pro-fibrotic factors, such as transforming growth factor β (TGF-β) and platelet-derived growth factor (13, 14). This cytokine network creates a pro-fibrotic environment where TGF-β, produced by M2 macrophages, serves as a master regulator of fibrosis by inducing fibroblast differentiation into myofibroblasts. In addition, T cells contribute to the inflammatory response and subsequent fibrosis process through immune regulation, while activated B cells release various cytokines and metalloproteinases, leading to dysregulation during the resolution phase of inflammation and excessive extracellular matrix deposition (6). Therefore, the progression of pulmonary fibrosis involves both inflammatory responses and immunologic mechanisms.

NOD-like receptor (NLR) family pyrin domain-containing 3 (NLRP3) inflammasome is a cytoplasmic multiprotein complex that plays a critical role in regulating inflammation and immune responses (15, 16). It is not only present in macrophages but also in epithelial cells and myofibroblasts (17, 18). Its primary function is to recognize pathogen-associated molecular patterns (PAMPs) and damage-associated molecular patterns (DAMPs), initiating innate immune responses, activating inflammatory reactions, and promoting the secretion of IL-1β and IL-18, which further drive downstream pathways and molecular changes (19). The NLRP3 inflammasome plays a dual role in various diseases, protecting the host from infection while potentially causing tissue damage under pathological conditions such as chronic inflammation and fibrosis. Increasing evidence suggests that activation of the NLRP3 inflammasome is closely associated with the progression of pulmonary fibrosis. Studies on the genetic polymorphism of pulmonary fibrosis have found that the NLRP3 *rs35829419* variant allele is associated with an increased risk of asbestos-related PF (20). Elevated levels of NLRP3 have been observed in the bronchoalveolar lavage fluid (BALF) of rheumatoid arthritis-associated interstitial lung disease (RA-ILD) patients (21). Aberrant activation of the NLRP3 inflammasome has also been detected in animal models of pulmonary fibrosis induced by PM2.5, silica dust, asbestos, and BLM (8, 22–25). Moreover, inhibition of the NLRP3 inflammasome has been shown to attenuate the progression of pulmonary fibrosis (26).

Therefore, the NLRP3 inflammasome not only plays a role in inducing inflammatory responses and promoting the fibrotic process in pulmonary fibrosis, but it may also be an effective target for the treatment of pulmonary fibrosis. This review systematically summarizes the role and related mechanisms of the NLRP3 inflammasome in pulmonary fibrosis and integrates the latest research progress on NLRP3 inflammasome-targeted therapies in pulmonary fibrosis, hoping to provide new ideas and directions for clinical treatment.

## 2 The structure and activation of NLRP3 inflammasome

### 2.1 Inflammasome structure and assembly

The NLRP3 inflammasome is a sophisticated multiprotein complex with distinct structural components that determine its function. The NLRP3 inflammasome consists of NLRP3 protein, apoptosis-associated speck-like protein (ASC), and precursor of cysteine-aspartic protease-1 (pro-caspase-1) (27). Structurally, NLRP3 is characterized by a C-terminal leucine-rich repeat (LRR) domain, a central nucleotide oligomerization domain (named NACHT), and an N-terminal pyrin domain (PYD) (28). The NACHT domain includes a nucleotide-binding domain (NBD) and a winged-helix domain positioned between two helical domains (designated HD1 and HD2) (28, 29). The NBD contains highly conserved Walker A and Walker B motifs essential for ATP binding and hydrolysis, respectively (30).

The adaptor protein ASC contains both a PYD and a caspase activation and recruitment domain (CARD), allowing it to bridge NLRP3 and pro-caspase-1 through homotypic domain interactions. Upon activation, NLRP3 oligomerizes and interacts with ASC via PYD-PYD interactions, leading to ASC oligomerization into a large speck-like structure. This ASC speck serves as a platform that recruits multiple pro-caspase-1 molecules through CARD-CARD interactions, facilitating proximity-induced auto-activation of caspase-1 (11, 16).

Research has identified NIMA-related kinase 7 (NEK7) as a critical binding partner that directly interacts with NLRP3 to mediate inflammasome assembly and activation (31–33). This interaction occurs in a cell cycle-dependent manner, linking inflammasome activation to the cell cycle status. The NEK7-NLRP3 interaction is required downstream of potassium efflux, suggesting that NEK7 functions as a switch that permits NLRP3 activation only under specific cellular conditions.

### 2.2 Inflammasome activation

As the core sensor of the inflammasome, NLRP3 activation generally requires two signals: the first signal is the initial activation through pattern recognition receptors (PRRs), such as Toll-like receptors (TLRs), leading to NF-κB-mediated upregulation of NLRP3 and pro-IL-1β expression. This priming step not only increases the expression of inflammasome components but also induces post-translational modifications of NLRP3 that license its activation. The second signal involves diverse cellular stressors that converge on common pathways to trigger NLRP3 conformational changes and assembly. Key activation factors include:

#### 2.2.1 Ionic flux

Potassium efflux represents a universal trigger for NLRP3 activation across diverse stimuli (34). Experimental evidence shows that elevated extracellular potassium concentrations inhibit NLRP3 activation, while agents that induce potassium efflux, such as ATP and nigericin, are potent NLRP3 activators. Calcium signaling also plays a crucial role, with calcium mobilization from both extracellular spaces and intracellular stores contributing to inflammasome activation. Additionally, chloride efflux acts as a specific signal for ASC oligomerization and inflammasome assembly (35, 36).

#### 2.2.2 Mitochondrial dysfunction

Damaged mitochondria release mitochondrial reactive oxygen species (mtROS), mitochondrial DNA, and cardiolipin, which can directly or indirectly activate NLRP3 (8, 37). The mitochondria-associated ER membrane (MAM) serves as a platform for NLRP3 recruitment and activation, highlighting the interorganelle communication in inflammasome regulation (38).

#### 2.2.3 Lysosomal damage

Particulate matter such as crystalline silica, asbestos, and PM2.5—all implicated in pulmonary fibrosis—can destabilize lysosomes, leading to the release of lysosomal contents including cathepsins that activate NLRP3 (39–41).

In addition, some studies have shown that the cytoskeleton and Golgi apparatus are also involved in the activation process of NLRP3, but the specific mechanism remains to be explored (42–44). Upon activation, NLRP3 binds to ASC via PYD-PYD interactions. Multiple ASC molecules then oligomerize into speck-like structures, and ASC further recruits pro-caspase-1 into a complex through CARD-CARD interactions. Studies have demonstrated that chloride efflux acts as a signal for ASC oligomerization and plays a pivotal role in NLRP3 inflammasome assembly (35, 36). Additionally, NLRP3 activation is regulated by multiple post-translational modifications, such as SUMOylation and ubiquitination, which affect NLRP3 stability and function, subsequently influencing inflammasome activation. Ubiquitination typically inhibits NLRP3 by promoting its degradation, while deubiquitination by enzymes like BRCC3 is required for activation. Phosphorylation can either activate or inhibit NLRP3 depending on the specific residues modified and the kinases involved. SUMOylation of NLRP3 maintains it in an inactive state, and deSUMOylation is necessary for activation.

## 3 The NLRP3 inflammasome as a central node connecting inflammation and fibrosis in PF

**Figure 1:**
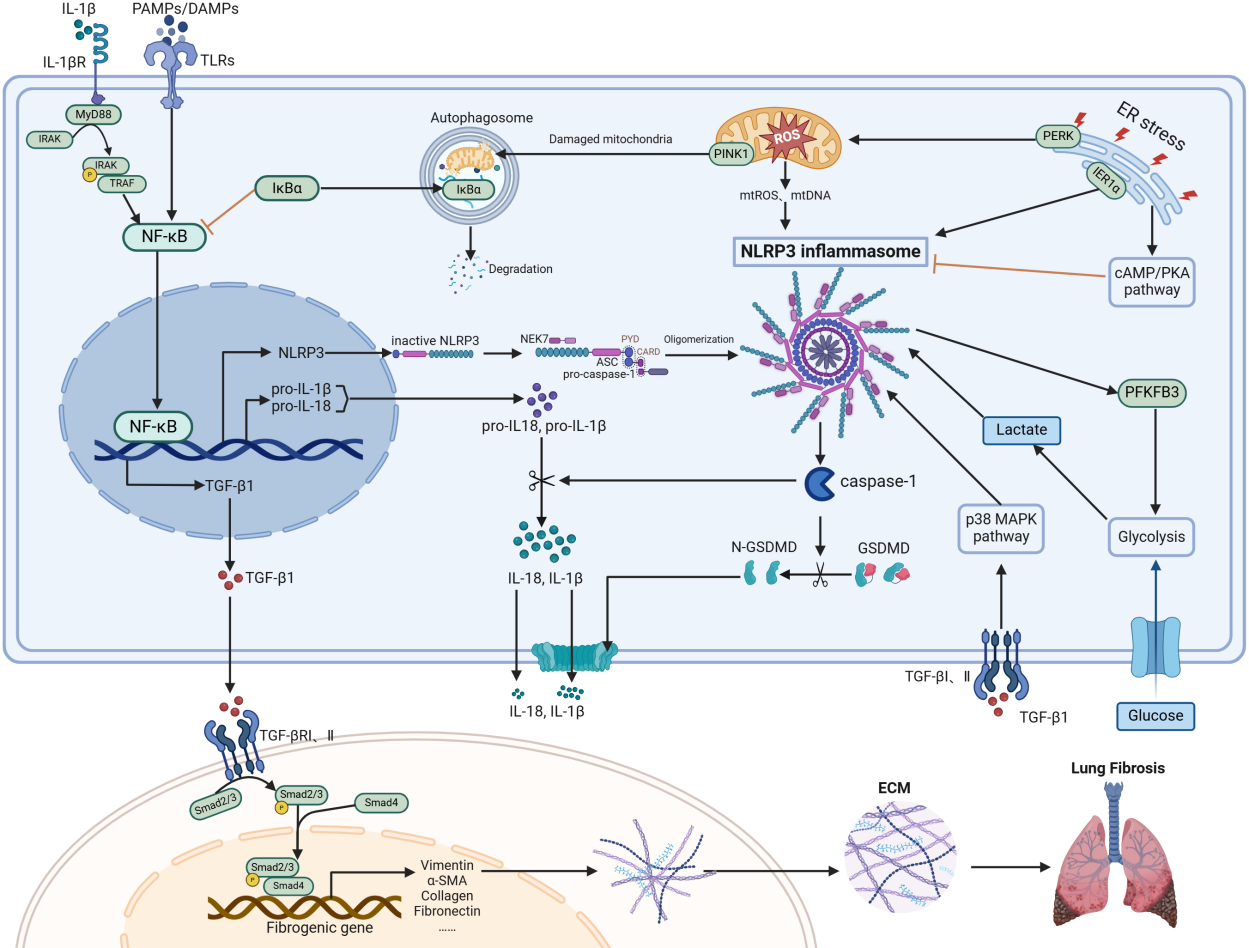
Regulatory mechanisms of NLRP3 inflammasome in PF. The NLRP3 inflammasome, mainly consisting of NLRP3, ASC, and pro-caspase-1, is supplied with components by the NF-κB signaling pathway. This complex can be activated in various cell types, particularly macrophages and type II alveolar epithelial cells. Activation of the inflammasome causes caspase-1 activation, leading to the maturation and release of IL-1β/IL-18 and GSDMD-mediated pyroptosis. A positive feedback loop between the NLRP3 inflammasome and NF-κB is established through IL-1β. ER stress, oxidative stress, autophagy, and metabolic changes such as glycolysis are all involved in regulating the levels of the NLRP3 inflammasome. TGF-β1 and NLRP3 inflammasome mutually enhance each other, collectively driving the progression of pulmonary fibrosis by promoting fibroblast differentiation into myofibroblasts and subsequent ECM accumulation. Figure was created with biorender.com.

Although the precise pathogenesis of pulmonary fibrosis remains unclear, substantial research has highlighted the involvement of key signaling pathways and biological mechanisms. As the NLRP3 inflammasome is investigated, its role in the progression of pulmonary fibrosis is progressively being elucidated. In the progression of pulmonary fibrosis, various cell types participate in disease development through the NLRP3 inflammasome. Type II alveolar epithelial cells (ATII) are pivotal both for NLRP3 expression and fibrotic progression; under endoplasmic reticulum stress and oxidative stress conditions, NLRP3 activation in ATII cells leads to IL-1β production and pyroptosis (45), while epithelial-mesenchymal transition (EMT) of ATII cells directly promotes fibrosis (46). Alveolar macrophages, as the predominant immune cells in the lungs, represent a major source of NLRP3 inflammasome activation and IL-1β production (14), with their polarization state (M1/M2) being regulated by NLRP3 and subsequently influencing the fibrotic process (47, 48). The following sections will delve into the complex interactions between the NLRP3 inflammasome and various signaling pathways and cellular mechanisms in pulmonary fibrosis (**Figure 1**).

### 3.1 The NLRP3 inflammasome and inflammatory signaling cascades: NF-κB/NLRP3/IL-1β pathway

In the initial stage of lung injury caused by environmental factors, bacterial and viral infections, and drugs that lead to the damage of epithelial cells and cause the inflammation of alveolar macrophages. PRRs recognize PAMPs and DAMPs to initiate the innate immune response, activating the NF-κB signaling pathway. Then, the expression of NLRP3, pro-IL-1β, and pro-IL-18 were upregulated, providing the necessary components for inflammasome activation (49). Subsequently, variable factors such as potassium efflux, calcium influx, mitochondrial reactive oxygen species (ROS) generation, and lysosomal disruption may trigger the assembly and activation of the NLRP3 inflammasome (50–52). After the NLRP3 inflammasome is activated, active caspase-1 further cleaves pro-IL-1β and pro-IL-18 into mature IL-1β and IL-18, which are then released (51). IL-1β, as a pro-inflammatory cytokine, plays a multifaceted role in the formation of pulmonary fibrosis. Early research indicates that IL-1β promotes pulmonary fibrosis *in vivo* by activating IL-1 receptor-dependent signaling pathways (11). Additionally, preclinical models of pulmonary fibrosis show that the expression levels of MyD88 in lung tissue increase after exposure to various profibrotic stimuli (53). Studies have demonstrated that the activated NLRP3 inflammasome can promote pulmonary EMT via the IL-1β/IL-1Rs/MyD88/NF-κB signaling pathway, leading to pulmonary fibrosis (14). After IL-1β binds to the IL-1R1 receptor on the cell surface, it recruits MyD88 and activates the IL-1 receptor-associated kinases (IRAK). These kinases, once phosphorylated, dissociate from MyD88 and bind to the tumor necrosis factor receptor-associated factor (TRAF), further inducing the activation of the transcription factor NF-κB (54). Then, the activated NF-κB upregulates the expression of NLRP3 and IL-1β again, forming a positive feedback loop that triggers an inflammatory cascade, ultimately leading to pulmonary fibrosis (8, 55). Also, the activated NF-κB pathway drives the expression of pro-inflammatory factors such as TNF-α, IL-6, and chemokines (like IL-8), resulting in an amplified inflammatory effect (56). In mice with IL-1R1 and MyD88 gene knockout, BLM administration did not provoke a fibrotic response (8). It can be seen that in the process of pulmonary fibrosis, the NF-κB signaling pathway is not only the initiating signal for the activation of the NLRP3 inflammasome but also has a positive feedback mechanism with NLRP3, leading to the persistent progression of pulmonary fibrosis. The activation of NF-κB not only directly promotes the expression and activation of the NLRP3 inflammasome, but may also be regulated by various cellular stress responses, which play important roles in the occurrence and development of pulmonary fibrosis.

### 3.2 The upstream regulatory factors of the NF-κB/NLRP3/IL-1β pathway in PF: the oxidative stress, ER stress, autophagy, and metabolism

#### 3.2.1 Oxidative stress

Oxidative stress results from an imbalance between the production of oxidants and the cell’s antioxidant capacity, leading to the generation of ROS including hydrogen peroxide (H_2_O_2_), superoxide anion (O^2-^), hydroxyl radical (OH), hypochlorous acid (HOCl), and peroxynitrite (ONOO) (57). Mitochondrial dysfunction is associated with oxidative stress and releases signals such as excessive mtROS, translocation of mtDNA to the cytoplasm, or repositioning of mitochondria, all of which are considered direct activators of the NLRP3 inflammasome (58). In pulmonary fibrosis, excessive production of ROS can trigger the death of phagocytic cells, recruitment of inflammatory cells, and permanent lung damage. Previous studies have shown that the interaction between fibrogenic inducers and pulmonary macrophages stimulates the production of a large amount of ROS and activates the intracellular NF-κB signaling pathway, leading to the activation of the NLRP3 inflammasome and the secretion of IL-1β. IL-1β further promotes the transformation of fibroblasts and may in turn increase the production of ROS, leading to the continuous progression of pulmonary fibrosis (59). Early research has proven that antioxidant regulator Nrf2 is one of the key downstream mechanisms involved in mitochondrial dysfunction (60). It not only links to the expression of many genes required for mitochondrial respiratory function but also plays critical roles in enhancing mtDNA levels, oxidative phosphorylation (OXPHOS) activity, and mitochondrial protein import and assembly (61). In the IL-33 and LPS/IL-4-induced pulmonary fibrosis cell model, ROS production and mtDNA accumulation, key upstream events of NLRP3 activation in macrophages, were observed, along with a reduction in Nrf2 mRNA levels. When Nrf2 expression is absent, over-activation of NLRP3 leads to the release of a large amount of pro-inflammatory factors such as IL-1β and IL-18 (13). During another study, oxidative stress-associated protein high mobility group box 1 (HMGB1) can activate NLRP3 inflammasomes and promote PF by inhibiting the Nrf2/HO-1 pathway in BLM-induced PF model (62). Additionally, studies have shown that enhanced ROS levels can exacerbate the development of PF through NLRP3-mediated cellular senescence, and that the acceleration of PF by ROS is positively correlated with cellular senescence (63).

#### 3.2.2 ER stress

The ER plays a crucial role in maintaining protein homeostasis (64). However, factors such as aging, hypoxia, oxidative stress, or inflammation can disrupt this balance, leading to the accumulation of misfolded proteins in ER, which activate the unfolded protein response (UPR), and thereby triggering ER stress and apoptosis (65). Relevant studies have shown that ER stress plays a key role in the occurrence and progression of pulmonary fibrosis (66, 67). ER stress affects cellular behavior and function through abnormal activation of UPR signaling pathways, including inositol-requiring enzyme 1 (IRE1), protein kinase R-like endoplasmic reticulum kinase (PERK), and activating transcription factor 6 (ATF6) signaling pathways, which lead to cell apoptosis, EMT, fibroblast differentiation, and macrophage polarization (65). A substantial amount of research indicates that NLRP3 is involved in ER stress within ATII cells during pulmonary fibrosis. In ATII cells of patients with IPF, a large accumulation of unfolded or misfolded proteins has been observed. These abnormal proteins dephosphorylate the ER transmembrane receptor IRE1α. The dephosphorylation of IRE1α can further activate the NLRP3 inflammasome, thereby promoting the expression of IL-1β and IL-18 (68). Additionally, some studies have found that sustained ER stress activates the PERK pathway, disrupts mitochondrial homeostasis, and leads to the release of mitochondrial damage-associated molecular patterns (mt-DAMPs). These mt-DAMPs (such as mitochondrial DNA and ROS) activate the NLRP3 inflammasome either within the cell or outside the cell, further driving the release of profibrotic factors (such as IL-1β and IL-18) (69). Simultaneously, *in vitro* pulmonary fibrosis models have shown that ER stress promotes the activation of the cAMP/PKA pathway, which may inhibit the activation of the NLRP3 inflammasome in AECs II induced by ER stress. Therefore, the cAMP/PKA pathway has a certain protective effect against pulmonary fibrosis (45).

Cellular stress including oxidative stress and ER stress not only directly affects the activation of the NLRP3 inflammasome but may also indirectly influence inflammasome function by regulating the autophagy process, forming a complex regulatory network (70).

#### 3.2.3 Autophagy

Autophagy is a lysosome-dependent cellular self-degradation process that plays a critical role in clearing damaged or excess organelles and proteins, thereby maintaining homeostasis within the organism (71, 72). Increasing evidence suggests that autophagy is closely associated with the process of pulmonary fibrosis. For instance, exposure to silica nanoparticles activates autophagy, ultimately leading to endothelial dysfunction in silicosis (73). Another study found that insufficient autophagy results in the senescence of lung epithelial cells and the differentiation of fibroblasts into myofibroblasts in IPF (74). In an LPS-induced pulmonary fibrosis model, impaired autophagy was observed alongside NLRP3 inflammasome activation. Treatment with oridonin significantly inhibited the activation of the NLRP3 inflammasome and reversed autophagy levels (75). In a PM2.5-induced pulmonary fibrosis model, PM2.5 was shown to downregulate ALKBH5 expression, promote m6A modifications at specific sites of *Atg13* mRNA, and activate ULK complex (composed of ULK1, Atg13, FIP200, and Atg101)-mediated autophagy. This autophagy further mediated the degradation of IκB-α (a NF-κB inhibitor), allowing NF-κB to translocate into the nucleus, which subsequently promoted the expression of NLRP3 and its downstream inflammatory factors (23). Moreover, studies have demonstrated that autophagy interacts with the pyroptosis pathway through the NLRP3 inflammasome. In the silica-induced fibrosis model, PTEN-induced kinase 1 (PINK1)-mediated mitophagy facilitates the clearance of damaged mitochondria, thereby negatively regulating NLRP3 inflammasome-associated pyroptosis. When inhibitors targeting NLRP3, caspase-1, and GSDMD were used to restrict the pyroptotic cascade, mitochondrial autophagy was enhanced (76). In addition to autophagy regulating NLRP3 inflammasome activity, metabolic alterations can also modulate NLRP3 activity.

#### 3.2.4 Metabolism

In recent years, the metabolic alterations associated with pulmonary fibrosis have garnered growing recognition and academic interest (77). In the lung tissue of IPF patients, 25 metabolic features have been identified, which suggest alterations in metabolic pathways, including glycolysis, glutathione biosynthesis, adenosine triphosphate (ATP) degradation, and ornithine transaminase pathways (78). Current research has found that the NLRP3 inflammasome is involved in metabolic abnormalities associated with PF. In lung tissue from a silica dust-induced PF model, overexpression of 6-phosphofructo-2-kinase/fructose-2,6-bisphosphatase 3 (PFKFB3) and an increase in lactate have been observed (7). PFKFB3 is a key enzyme in glycolysis, and lactate is a crucial substrate for histone lactylation. Both of them are involved in the process of fibrosis (79–81). In the activation of fibroblasts induced *in vitro*, the upregulation of PFKFB3 and an increase in extracellular lactate were also observed, along with the upregulation of the expression of NLRP3, ASC, and the activated caspase-1 p20 subunit. A selective NLRP3 inflammasome inhibitor MCC950 not only suppressed the activation of the NLRP3 inflammasome but also reduced the upregulation of PFKFB3 and the expression of fibrotic markers α-smooth muscle actin (α-SMA) and collagen I. At the same time, the study also found that lactate activated the NLRP3 inflammasome by increasing the level of histone lactylation (7). In another study, the glycolysis inhibitor (2-DG) alleviated CS-induced NLRP3 inflammasome activation and macrophage pyroptosis. Therefore, the activation of the NLRP3 inflammasome in fibroblasts is associated with glycolysis. Purine metabolism also participates in the progression of pulmonary fibrosis through the NLRP3 inflammasome. In BLM-induced PF model, uric acid released from injured cells activates the NLRP3 inflammasome, leading to IL-1β production. Reduction of uric acid levels using the inhibitor of uric acid synthesis allopurinol or uricase leads to a decrease in BLM-induced IL-1β production, lung inflammation, repair, and fibrosis (82). In addition, other studies have found that disorders in lipid metabolism may trigger NLRP3 inflammasome-related inflammatory cascade reactions, but the exact mechanism remains to be further studied (83). Metabolic alterations promote the activation of the NLRP3 inflammasome, which further induces pyroptosis, releases inflammatory cytokines, and forms a pro-fibrotic microenvironment.

### 3.3 The role of pyroptosis mediated by NLRP3 inflammasome in PF

Pyroptosis is a type of cell death that triggers an inflammatory response and plays an important role in PF (84). The NLRP3 inflammasome plays a significant role in the initiation of pyroptosis (50, 77). Upon activation, the NLRP3 inflammasome drives the activation of caspase-1. Once activated, caspase-1 not only processes pro-IL-1β and pro-IL-18 into their biologically active forms but also cleaves gasdermin D (GSDMD), inducing the oligomerization of its N-terminal fragments (GSDMD-NT). These fragments assemble into 21-nanometer pores within the plasma membrane, facilitating the extracellular release of IL-1β and IL-18, compromising membrane integrity, and ultimately orchestrating the execution of pyroptosis (85). The intense inflammatory environment triggered by pyroptosis can activate myofibroblasts, leading to pulmonary fibrosis (83). Studies have shown that NLRP3-mediated pyroptosis in PF is closely related to TLR9 (86), which is an important member of the TLR family that primarily recognizes DNA containing unmethylated CpG sequences and plays a significant role in the pathogenesis of various diseases (87–89). In pulmonary fibrosis, self-DNA released from damaged cells (such as mitochondrial DNA and chromatin DNA) can activate TLR9 (88). Activated TLR9 promotes NF-κB activation, increasing NLRP3 and pro-IL-1β expression (88). Simultaneously, TLR9 signaling also promotes ROS production (90) and calcium ion change (91), thereby facilitating NLRP3 inflammasome assembly and activation. In mice with BLM-induced PF, it has been found that TLR9 expression is elevated, which increases the activation of the NLRP3/caspase-1 inflammasome pathway, thereby promoting pyroptosis of alveolar epithelial cells. In TLR9 knockdown mice, pulmonary fibrosis was alleviated and cellular pyroptosis was reduced. However, treatment with an NLRP3 activator reversed the levels of fibrosis and pyroptosis in the lung tissue of TLR9 knockout mice (86). Additionally, TLR9 can indirectly influence the activation state of the NLRP3 inflammasome by regulating the inflammatory responses of dendritic cells and macrophages (92). The cell death and more cytokine release mediated by pyroptosis not only directly promote inflammatory responses but also enhance TGF-β1 expression through augmentation of the NF-κB pathway, thereby driving the fibrotic process.

### 3.4 The NLRP3 inflammasome promote fibrotic signaling: TGF-β1 pathway

TGF-β1 is considered as a key mediator that drives the differentiation of fibroblasts into myofibroblasts and induces the expression of fibrosis-related genes (10). Elevated levels of TGF-β1 are detected in the BALF and lung tissue of IPF patients (93). Additionally, a genetic link has been discovered, with polymorphisms in the TGF-β1 gene associated with an increased susceptibility to IPF (94). Research has found that TGF-β1 is involved in the process of pulmonary fibrosis by activating both the canonical (SMAD-dependent) and non-canonical (SMAD-independent) pathways, with the SMAD signaling pathway being the primary one (51, 95). In the canonical pathway, active TGF-β is released and binds to the TGF-β type II receptor (TβRII), recruiting the TGF-β type I receptor (TβRI). TβRI is activated under the phosphorylation of TβRII, initiating kinase activity, which further leads to the phosphorylation of R-Smads (Smad2 and Smad3) (96). There is an evidence to suggest that the phosphorylation of Smad2/3 is a key profibrotic signal mediated by TGF-β1 (8). Activated Smad2 and Smad3 form a complex with Co-Smad (Smad4) and were translocated into the nucleus to regulate the expression of multiple profibrotic genes (97), promoting the transdifferentiation of fibroblasts into myofibroblasts and EMT (98). The non-canonical pathways include p38 mitogen-activated protein kinase (p38 MAPK), c-Jun N-terminal kinase (c-JNK), phosphatidylinositol 3-kinase (PI3K)-Akt-mTOR, NF-κB, transforming growth factor-β activated kinase 1 (TAK1), Janus kinase 2 (JAK2), signal transducer and activator of transcription 3 (STAT3), Raf-MEK1/2-ERK1/2, and Rho-associated kinase (ROCK) (95). These non-canonical pathways, in conjunction with the canonical pathway, promote the occurrence of fibrosis at multiple levels.

Recent studies have shown that NLRP3 inflammasome plays an important role in the fibrotic process induced by TGF-β1 (8). Compared to the control group, the levels of TGF-β1, phosphorylated Smad2/3 proteins, and the mRNA levels of NLRP3, caspase-1, and ASC in the lung tissues of mice exposed to BLM were all significantly increased. Concurrently, *in vitro* experiments found that the upregulation of NLRP3 reversed the inhibition of the TGF-β1/Smad2/3 pathway (51). Similarly, in a rat model exposed to silica, the expressions of NLRP3, TGF-β1, and IL-1β were all increased, and the activated NLRP3 inflammasome promoted the secretion of inflammatory cytokines IL-1β and TGF-β (10). Furthermore, it was found that after silencing the expression of NLRP3 *in vitro*, the protein level of TGF-β1 was significantly reduced (26). Early studies have found that in lung cells (A549), IL-1β stimulates TGF-β1 transcription through temporal regulation by NF-κB and AP-1 (99). As noted earlier, NF-κB plays a pivotal role in the assembly and activation of the NLRP3 inflammasome as well as the production of IL-1β. Therefore, it is plausible that the NLRP3 inflammasome promotes TGF-β1 expression via the NF-κB signaling pathway. The exact regulatory mechanisms in pulmonary fibrosis remain to be further investigated. In another study, to simulate intercellular interactions in pulmonary fibrosis, alveolar epithelial cells and macrophages were co-cultured under hypoxic conditions. This co-culture resulted in increased expression of NLRP3, TGF-β1, and TGFBRs, alongside a significant downregulation of SMAD7, a negative regulator of TGF-β signaling, thereby promoting EMT. Notably, silencing TGF-β1 or applying TGF-β1 inhibitors, TβRI kinase inhibitors, or p38 MAPK inhibitors effectively suppressed the upregulation of NLRP3 expression. However, treatment with a SMAD3 phosphorylation inhibitor did not lead to significant changes in NLRP3 expression levels. These findings indicate that TGF-β1 regulates NLRP3-mediated EMT primarily through the p38 MAPK pathway rather than the SMAD3 phosphorylation pathway (100).

In conclusion, the role of NLRP3 inflammasome in PF constitutes a comprehensive signaling network. This begins with PAMPs/DAMPs-mediated NF-κB activation as the upstream trigger, continues through cellular stress (oxidative stress and ER stress), autophagy, and metabolic reprogramming as regulatory mechanisms, and proceeds to NLRP3-mediated pyroptosis which serves as a positive feedback amplifying inflammatory responses. Ultimately, the NLRP3 inflammasome enhances the TGF-β1 signaling pathway, leading to ECM deposition and tissue fibrosis. This complete signaling cascade demonstrates the NLRP3 inflammasome as a central node in the pathological process of pulmonary fibrosis, connecting inflammatory responses and fibrotic progression, thus providing a theoretical basis for targeted therapeutic strategies.

## 4 Targeting NLRP3 inflammasome for the treatment of PF

As mentioned above, NLRP3 inflammasome plays a critical role in the pathological mechanism of PF. Therefore, targeting the NLRP3 inflammasome for the treatment of PF has become one of the current research hotspots. Several drugs have been developed to inhibit NLRP3 inflammasome in different disease models, such as ZYIL1, DFV890, VTX(2735, 3232), emlenoflast, selnoflast, NT(0796, 0527, 0249), dapansutrile, tranilast, MCC950, 3,4-Methylenedioxy-β-nitrostyrene, CY-09, RRx-001, and HT-6184, some of which are currently undergoing clinical trials (17, 101). **Table 1** summarizes several drugs targeting NLRP3 inflammasome in PF models.

**Table 1:** Inhibitors targeting NLRP3 inflammasome and their mechanism in PF models.

Inhibitor	Target			Mechanism	Reference
	NLRP3	ASC	Caspase-1		
Tranilast	+	–	–	Inhibit NLRP3 inflammasome activation and pyroptosis	(102)
Lycorine	–	+	–	Inhibition of inflammatory cascade and pyroptosis	(103)
MCC950	+	–	–	Inhibit inflammation and reduce collagen deposition	(104)
VX-765	–	–	+	Inhibition of inflammation and pyroptosis	(105)
Z-YVAD-FMK	–	–	+	Inhibition of NLRP3 inflammasome and mitigation of EMT	(106)

+, The inhibitor directly targets or has a significant effect on this component; –, The inhibitor does not directly target or has no significant effect on this component.

Tranilast (N-(3’,4’-dimethoxycinnamoyl)-anthranilic acid), a tryptophan metabolite analog, binds to the NACHT domain of NLRP3 and disrupts the NLRP3-ASC interaction without affecting NLRP3-NEK7 binding (17, 101). It has been approved in South Korea and Japan for the treatment of asthma, keloids, and hypertrophic scars (102). *In vivo* and *in vitro* experiments demonstrate that tranilast protects against acute respiratory distress syndrome and early pulmonary fibrosis induced by smoke inhalation (107). In a case report of severe COVID-19 pneumonia with secondary pulmonary fibrosis, six months of tranilast treatment significantly improved lung fibrosis and respiratory function (102).

Lycorine, a natural alkaloid extracted from the amaryllidaceae family, was initially reported to inhibit the growth and cell division of yeasts, algae, and higher plants (108). In recent years, studies have demonstrated its antitumor, anti-inflammatory, antioxidant, and antifibrotic effects. In the BLM-induced pulmonary fibrosis model, lycorine could disrupt the interaction of NLRP3 with ASC by targeting the PYD domain at Leu9, Leu50, and Thr53, thereby inhibiting NLRP3 inflammasome activation and pyroptosis, ultimately ameliorating BLM-induced pulmonary fibrosis (103).

MCC950 (CP-456,773 or CRID3) is one of the most extensively studied highly selective NLRP3 inflammasome inhibitors. Its exact mechanism of action remains unclear, but it has been applied in animal models of various diseases, including pulmonary fibrosis (33). A study demonstrated that MCC950 inhibits NLRP3 activation by converting its active conformation into an inactive state (109). Another study showed that MCC950 directly targets the Walker B motif in the NACHT domain of NLRP3, thereby blocking ATP hydrolysis and inhibiting NLRP3 activation and inflammasome formation (110). In BLM-induced PF model, MCC950 treatment inhibited the NLRP3 inflammasome, alleviated alveolar hemorrhage and alveolitis, and reduced collagen fiber deposition, thereby improving pulmonary fibrosis (104).

VX-765 is a specific caspase-1 inhibitor that has been proven in animal models to alleviate Alzheimer’s disease, epilepsy, and cardiovascular diseases (111). In the silica-induced pulmonary fibrosis model, VX-765 reduced the expression of inflammatory cytokines, including IL-1β, TNF-α, IL-6, CCL2, and CCL3, downregulated endogenous DAMPs and inflammation-related pattern recognition receptors TLR4 and NLRP3, inhibited pyroptosis of alveolar macrophages, and decreased α-SMA, collagen, and fibronectin levels, thereby alleviating pulmonary fibrosis (105).

Z-YVAD-FMK, another caspase-1 inhibitor, has been utilized in various disease models, including ischemic stroke, neuroblastoma, epilepsy, myocardial infarction, and fibrosis (112–116). In a PM2.5-induced pulmonary fibrosis model, treatment with Z-YVAD-FMK effectively inhibited IL-1β secretion and the overexpression of ASC and NLRP3 proteins (18). Similarly, in a silicosis-induced pulmonary fibrosis model, Z-YVAD-FMK suppressed the activation of the NLRP3 inflammasome and mitigated silica-induced EMT (106).

Other drugs and molecules can also influence NLRP3 inflammasome in pulmonary fibrosis models. Nintedanib and pirfenidone both suppressed NLRP3 inflammasome activation in pulmonary fibrosis models, exerting anti-inflammatory effects (117, 118). Asiatic acid (AA), isolated from Centella Asiatica, has been shown to reduce NLRP3 expression in PF model, although the exact mechanism remains to be further investigated (119). Exosomes derived from mesenchymal stem cells and small RNA molecules may also alleviate pulmonary fibrosis by modulating the NLRP3 inflammasome (104, 120). In addition, several studies have identified potential targets, such as the autophagy adapter P62/SQSTM1, which can inhibit the excessive activation of the NLRP3 inflammasome by transporting ubiquitinated ASC to autophagosomes for degradation and through a positive feedback loop with Nrf2-ARE (121). The overexpression of membrane protein Caveolin-1 can suppress the NLRP3 inflammasome and its associated expression of interleukin-1β (IL-1β), thereby hindering the progression of fibrosis (122).

In preclinical studies of pulmonary fibrosis therapeutics, many laboratories utilize the bleomycin-induced pulmonary fibrosis mouse model (123). While mouse models, particularly the BLM-induced model, provide valuable tools for studying PF, it is important to note that these models exhibit significant differences from human IPF (124). The BLM model primarily represents fibrotic responses following acute lung injury, rather than the slowly progressive course characteristic of human IPF. The inflammatory response induced by BLM is typically more pronounced than that observed in human IPF (125). Additionally, species differences between mice and humans in NLRP3 regulation and inflammatory responses may affect the clinical translatability of research findings. Therefore, when evaluating NLRP3-targeted strategies, animal model data should be interpreted cautiously, and more clinically relevant experimental systems should be actively explored, such as humanized mouse models, human lung tissue organoids, and other approaches that more closely approximate human pathology.

## 5 Conclusions and future perspectives

PF is an interstitial lung disease with an unclear pathogenesis, presenting significant challenges for its treatment. The NLRP3 inflammasome has been extensively studied in various diseases, and its role in pulmonary fibrosis has been increasingly explored in recent years. This review discusses the role of the NLRP3 inflammasome in pulmonary fibrosis and its potential mechanisms. The NLRP3 inflammasome participates in inflammatory responses and immune regulation in the body, forms a positive feedback loop with the NF-κB pathway during the progression of pulmonary fibrosis, and interacts with the TGF-β1 signaling pathway to promote EMT, myofibroblast formation, and extracellular matrix accumulation, thereby driving fibrotic processes. Furthermore, the NLRP3 inflammasome is involved in the progression of PF through mechanisms such as oxidative stress, ER stress, pyroptosis, autophagy, and metabolism. Thus, the NLRP3 inflammasome is an important regulatory factor in PF. In addition, the potential of the NLRP3 inflammasome as a therapeutic target has gradually emerged. Several drugs targeting the NLRP3 inflammasome have already been developed, with some demonstrating promising therapeutic effects in pulmonary fibrosis models. However, additional in-depth research is required before these drugs can be translated into clinical applications. Consequently, future investigations should prioritize a comprehensive understanding of the mechanistic role of the NLRP3 inflammasome in pulmonary fibrosis, rigorously evaluate the efficacy and safety profiles of targeted therapies, and advance the development of personalized treatment strategies. Moreover, the integration of advanced drug delivery systems (DDS), including nano-delivery, hydrogel delivery, and biological carrier delivery, holds great potential in minimizing side effects and enhancing drug bioavailability (126). As such, the future application of DDS to specifically target the NLRP3 inflammasome could significantly improve the therapeutic outcomes in pulmonary fibrosis management.

Additionally, studies have revealed a close association between PF and lung cancer, with IPF patients exhibiting a 2.4-7.5-fold increased risk of developing lung cancer compared to the general population (127). The NLRP3 inflammasome appears to function as a molecular bridge in this transformation from IPF to cancer (103). Mechanistically, the chronic inflammatory microenvironment maintained by persistent NLRP3 activation promotes genomic instability and DNA damage (128, 129), while downstream inflammatory mediators such as IL-1β facilitate tumor cell proliferation and angiogenesis (130, 131). Furthermore, the extensively remodeled extracellular matrix and altered growth factor signaling characteristic of fibrotic tissues create a conducive microenvironment for tumor cell survival and migration. This dual involvement of the NLRP3 inflammasome in both IPF pathogenesis (14) and malignant transformation (132) positions it as a promising therapeutic target with potential to simultaneously address both serious pulmonary conditions.

In conclusion, the NLRP3 inflammasome provides a new perspective for understanding the pathogenesis of pulmonary fibrosis and offers novel therapeutic strategies for its treatment.
